# The association of asthma, atopic dermatitis, and allergic rhinitis with peripartum mental disorders

**DOI:** 10.1002/clt2.12082

**Published:** 2021-12-03

**Authors:** Tai Ren, Jiawen Chen, Yongfu Yu, Hua He, Jun Zhang, Fei Li, Katrine Svendsen, Carsten Obel, Hui Wang, Jiong Li

**Affiliations:** ^1^ Ministry of Education – Shanghai Key Laboratory of Children's Environmental Health Xinhua Hospital Shanghai Jiao Tong University School of Medicine Shanghai China; ^2^ Department of Clinical Medicine and Epidemiology Aarhus University Aarhus Denmark; ^3^ Department of Dermatology Xinhua Hospital Shanghai Jiao Tong University School of Medicine Shanghai China; ^4^ Department of Biostatistics Key Laboratory of Public Health Safety School of Public Health Fudan University Shanghai China; ^5^ Department of Developmental and Behavioural Paediatric & Child Primary Care Xinhua Hospital Shanghai Jiao Tong University School of Medicine Shanghai China; ^6^ Department of Public Health Aarhus University Aarhus Denmark

**Keywords:** allergic rhinitis, asthma, atopic dermatitis, epidemiology, peripartum mental disorders, asthma, atopische dermatitis, allergischer schnupfen, epidemiologie, peripartale psychische störungen

## Abstract

**Background:**

Atopic diseases are characterized by dysregulated inflammatory response, which may incur the onset of peripartum mental disorders, but the impact remains unknown. This study examined whether and to what extent the history of atopic diseases is associated with newly onset peripartum mental disorders.

**Methods:**

Using population‐based registries, we identified all primiparous women who gave birth to live singletons in Denmark during 1978–2016 (*n* = 937,422). The exposure was hospital contact due to the three major types of atopic diseases—asthma, atopic dermatitis, and allergic rhinitis—before conception. The primary outcome was any hospital contact for mental disorder during pregnancy and 1‐year postpartum, which was further classified into affective disorders, neurotic, stress‐related and somatoform disorders, and substance abuse. The follow‐up started from the date of conception and ended at the date of the first diagnosis of mental disorders, 1‐year postpartum, death, emigration, or December 31, 2016, whichever came first. Cox regression was used, adjusted for calendar year, age at childbirth, education, residence, and Charlson comorbidity index.

**Results:**

A total of 24,016 (2.6%) women received diagnosis of at least one of the three atopic diseases before conception (asthma, 1.7%; atopic dermatitis, 0.6%; and allergic rhinitis, 0.8%). Exposure to asthma, atopic dermatitis, or allergic rhinitis was associated with a 37% increased overall risk of peripartum mental disorders (hazard ratio [HR], 1.37; 95% confidence interval [CI], 1.27–1.49). Higher risks were observed among women with more frequent hospital contacts for atopic disease (HR, 1.80; 95% CI, 1.37–2.35; ≥5 times), and with recent hospital contacts for atopic disease (HR, 1.74; 95% CI, 1.48–2.06; within 2 years before conception). Specific associations were observed between asthma and neurotic, stress‐related and somatoform disorders (HR, 1.40; 95% CI, 1.21–1.62), and between atopic dermatitis and substance abuse (HR, 1.62; 95% CI, 1.12–2.34).

**Conclusions:**

History of asthma, atopic dermatitis, and allergic rhinitis before conception was associated with increased risks of peripartum mental disorders. Women who have atopic diseases before pregnancy may benefit from systematic mental health monitoring.

## 1 INTRODUCTION

Peripartum period is a distinct and significant period of neural plasticity for females, driven by changes in hormones and immune molecules, resulting in increased susceptibility to mental disorders.^1^, ^2^ Peripartum mental disorders affect over 10% of pregnant and postpartum women, associated with both adverse pregnancy outcomes in mothers and behavioral problems in children.^3^, ^4^, ^5^ Though the underlying mechanisms remain to be elucidated, peripartum mental disorders have been related to several immune‐related disorders, such as inflammatory bowel disease, pre‐eclampsia, and gestational diabetes.^6^, ^7^, ^8^

Atopy is a personal or familial propensity to produce IgE antibodies in response to environmental triggers, contributing to atopic diseases.^9^, ^10^ Atopic dermatitis, asthma, and allergic rhinitis are the most common atopic diseases that affect approximately 20% of the population worldwide, and often develop sequentially, known as the “atopic march”.^9^, ^10^ The shared pathogenesis of dysregulated inflammatory response has been reported to trigger the onset of depression and anxiety in the general population.^11^, ^12^, ^13^, ^14^ The findings on the association between asthma and postpartum depression have, however, been inconsistent.^15^, ^16^, ^17^ The prevalence of atopic diseases, mostly developed before childbearing ages, is on the increase.^18^ To the best of our knowledge, the association of atopic dermatitis and allergic rhinitis with peripartum mental disorders has not been studied. Moreover, while most studies investigated risk factors for postpartum‐onset mental disorders,^19^ the *Diagnostic and Statistical Manual of Mental Disorders, Fifth Edition* (*DSM‐5*) has adopted the definition of peripartum mental disorders, emphasizing that pregnancy‐related mental disorders onset both during pregnancy and postpartum.^20^ In this population‐based cohort study using data from Danish national registries, we aimed to evaluate the association between the three atopic diseases (asthma, atopic dermatitis, and allergic rhinitis) before conception and newly onset peripartum mental disorders in pregnant women.

## 2 METHODS

### 2.1 Study population and follow‐up

We studied all primiparous women, who gave birth to a live singleton from 1978 to 2016 in Denmark, recorded in the Danish Medical Birth Registry (*n* = 1,045,372), which prospectively collects data from pregnancy to the peripartum period for approximately 99% of births in Denmark.^21^ We excluded pregnancies with unknown sex of offspring (*n* = 445), unrealistic gestational age (<154 or >315 days, *n* = 256), unknown conception date (*n* = 35,121), and mental disorder diagnosed before conception (any discharge diagnosis of *The International Classification of Diseases*, *Eighth Revision* [ICD‐8] codes 290–315, and *The International Statistical Classification of Diseases and Health Related Problems, Tenth Revision* [ICD‐10] codes F00–F99 from the Danish Psychiatric Central Research Register; *n* = 67,209). We also excluded women diagnosed with allergic conjunctivitis before conception (any discharge diagnosis of ICD‐10 code H10, available after 1995; *n* = 4919) in the unexposed group (as shown in the Exposure section) because of the shared etiology of atopy with the three atopic diseases of interest. The follow‐up period started from the conception date, and ended on the date of a first diagnosis of any mental disorder, death, emigration, 12 months after birth, or end of follow‐up (December 31, 2016), whichever came first. A total of 7148 (0.8%) women emigrated or died during the follow‐up time.

The study was approved by the Danish Data Protection Agency (Record No. 2013‐41‐2569). By Danish law, no informed consent is required for a registry‐based study using anonymized data.

### 2.2 Exposure

The unique civil registration number assigned to all Danish residents permits accurate linkage of individual‐level data from different national registers.^21^ The diagnosis of atopic diseases was retrieved from the Danish National Patient Register, which has recorded information on all patients discharged from Danish nonpsychiatric hospitals since 1977, and on emergency and outpatient contacts since 1995. The Patient Register contains no data on visits to general practitioners. The diagnoses were coded according to from 1969 through 1993, and ICD‐10 since 1994. We defined the exposure as the diagnosis of either asthma (ICD‐8 code 493 and ICD‐10 code J45–J46), or atopic dermatitis (ICD‐8 code 691 and ICD‐10 code L20), or allergic rhinitis (ICD‐8 code 507 and ICD‐10 code J30) before conception.^13^, ^22^, ^23^. Atopic multimorbidity was defined as the diagnosis of more than one type of the three atopic diseases.^10^ The frequency of atopic disease hospital contacts before conception was used as a proxy for recurrent atopic diseases (1, 2–5, or ≥5).^24^ The period from the last hospital contact of atopic diseases to conception was used as a proxy for disease activity (<2, 2–10, or ≥10 years).^25^ As the pathogenesis may differ between childhood‐ and adult‐onset atopic diseases, we categorized the exposed women into three groups based on their age at the first diagnosis of atopic disease (<10, 10–20, or >20 years old).^26^, ^27^, ^28^ Considering seasonality may influence the severity of atopic diseases, we categorized the exposed women according to the season of childbirth (winter, December–February; spring, March–May; summer, June–August; autumn, September–November).^29^

### 2.3 Outcome

The diagnosis of mental disorders was retrieved from the Danish Psychiatric Central Research Register, which contains every psychiatric inpatient since 1970 and outpatient and emergency room contacts since 1995.^21^ Peripartum mental disorders were defined as a newly onset mental disorder from conception date to the first‐year postpartum, coded as 290–315 in ICD‐8 and F00–F99 in ICD‐10 codes, in accordance with previous studies.^6^, ^7^, ^21^ Peripartum depression and anxiety represent the two most prevalent types of peripartum mental disorders.^30^ Thus, we were particularly interested in affective disorders (ICD‐8 codes 296.09 through 296.99, 298.19, 300.19, and 300.49; ICD‐10 codes F30 through F39), which were mainly comprised of peripartum depression, and neurotic, stress‐related and somatoform disorders (ICD‐8 codes 300.09 through 300.99 [except for 300.49], 305.09 through 305.99, 305.68, and 307.99; ICD‐10 codes F40 through F49), which were mainly comprised of peripartum anxiety.^31^, ^32^ We also analyzed substance abuse (ICD‐8 codes 291.xx, 303.xx, and 304.xx; ICD‐10 codes F10 through F19).^31^ Mental disorders unclassified in the former three clusters were classified as other mental disorders, mainly comprised of the following diagnostic entities: unspecified type of mental disorder (ICD‐10 code, F99), personality disorder (ICD‐8 code 301, ICD‐10 code, F60), and mental and behavioral disorders associated with the puerperium (ICD‐10 code, F53).

### 2.4 Covariates

Potential confounders were selected based on directed acyclic graphs (Figure S1), showing the best known relations between the variables in this study. Potential confounders included calendar period of delivery (≤1980, 5 ‐year intervals during 1981–2010, and 2011–2016), age at childbirth (<20, 20–24, 25–29, 30–34, or ≥35 years), education (0–9, 10–14, or ≥15 school years), residence (Copenhagen [approx. 800,000 inhabitants], cities with 100,000 or more inhabitants, or other places), and Charlson comorbidity index (0, or ≥1; detailed codes in Table S1). The Charlson comorbidity index was modified to exclude the disease categories related to our exposure (asthma in chronic pulmonary disease). As only 442 (0.05%) women had a Charlson comorbidity index ≥2, all women with a Charlson comorbidity index ≥1 were merged into a single category. Pregnancy complications and children characteristics include gestational hypertensive disorders (ICD‐8 codes 637.00, 637.02, 637.03, 637.04, 637.09, 637.19, 637.99, 639.99, 762.19, 762.29, 762.39, 762.99; ICD‐10 codes O12–O16; yes or no), gestational diabetes mellitus (ICD‐8 codes 634.74, ICD‐10 codes O24.4 and O24.9; yes or no), premature birth (gestational age <37 or ≥37 weeks), 5‐min Apgar score (<7, 7–9, or 10), low birth weight (<2500 or ≥2500 g), and newborn death (death within the first year of age; yes or no). Maternal smoking during pregnancy (yes or no) was only available after 1991, thus was only adjusted for in the sensitivity analyses.

### 2.5 Statistical analyses

Cox proportional hazards regression was applied to estimate the HRs and 95% CI, adjusted for the above‐mentioned potential confounders. The proportional hazard assumption was assessed by a log‐minus‐log plot (Figure S2). The two curves showed a roughly parallel pattern, and we found no obvious evidence supporting the interaction between HR and time (*p* for interaction 0.51). Thus, it's reasonable to accept the proportional hazard assumption. We evaluated risks of peripartum mental disorders in relation to the timing of diagnosis of mental disorder (during pregnancy or postpartum). Subgroup analyses were performed in the three types of atopic diseases. We used the likelihood‐ratio test to test for a trend in the analyses according to the atopy recurrence and atopy activity, with weights assigned according to the median of each category.^33^ A trend in atopy recurrence was assessed with weights of 1 in the group of women who had only one contact of atopic disease, 2 in the group two to four times, and 8 in the group five times or more. A trend in atopy activity was assessed with weights of 1 in the group of women whose latest contact of atopic disease before conception were within 2 years, 5 in the group 2–10 years, and 16 in the group more than 10 years.

Sensitivity analyses were performed as follows: (1) maternal smoking was further adjusted for in women giving birth after 1991 when the information on smoking was available; (2) to reduce the potential misclassification of the exposure, women with diagnoses of any of the three atopic diseases from conception to 2 years postpartum were excluded from the unexposed group; (3) because the coding system changed in 1994, women giving birth after 1994 were included in a separate analysis; (4) to test whether the association persists in multiparous settings, we identified all women giving birth to a live singleton from 1978 to 2016 in Denmark, with the same exclusion criteria described above, and randomly selected one pregnancy for multiparous women as the study population; (5) parturients with adverse pregnancy outcomes are often at higher risks of postpartum mental disorders, thus we restricted the analysis in women with favorable pregnancy outcomes (excluding offspring with 5‐min Apgar score <7, low birth weight, pre‐term birth, and neonatal mortality); (6) as the three atopic diseases might be diagnosed during pregnancy, the exposure was treated as a time‐varying variable during pregnancy; (7) the frequency of hospital contacts for each of the three atopic diseases was counted separately; (8) the test for trend was performed with different weights assigned to each category. In addition, Henriksen et al.^29^ reported an algorithm to identify atopic diseases with increased sensitivity by incorporating medication data from the Danish National Prescription Registry. A subanalysis was performed using the same algorithm with restriction on women giving birth to a live singleton between 1996 and 2016, when medication data were available (detailed methods were described in Appendix S1). All analyses were performed using Stata 15 and R version 3.6.1.

## 3 RESULTS

Of the 937,422 primiparous women giving birth to a live singleton between 1978 and 2016, 24,016 (2.6%) had received diagnosis of at least one of the three atopic diseases (asthma [1.7%], atopic dermatitis [0.6%], or allergic rhinitis [0.8%]) before conception; 4461 (18.6% of exposed women) had atopic multimorbidity (e.g., diagnoses of more than one type of the three atopic diseases). The women with asthma, atopic dermatitis, or allergic rhinitis were more likely to be older at pregnancy, have a high level of education, live in urban areas, and suffer from somatic comorbidities and pregnancy complications (Table 1).

**TABLE 1:** Baseline characteristics of primiparous women exposed and unexposed to asthma, atopic dermatitis, and allergic rhinitis before pregnancy in Denmark from 1978 to 2016

	No hospital contacts for atopic disease (*n* = 913,406)	Atopic disease (*n* = 24,016)
Maternal characteristics		
Age at childbirth (years)		
<20	43,016 (4.7)	937 (3.9)
20–24	251,316 (27.5)	5456 (22.7)
25–29	378,735 (41.5)	9973 (41.5)
30–34	183,099 (20.0)	5715 (23.8)
35+	57,240 (6.3)	1935 (8.1)
Education at childbirth (years)		
0–9	220,549 (24.1)	4445 (18.5)
10–14	416,279 (45.6)	10,712 (44.6)
15+	256,645 (28.1)	8798 (36.6)
Missing	19,933 (2.2)	61 (0.3)
Residence at childbirth		
Copenhagen (approx. 800,000 inhabitants)	133,200 (14.6)	4022 (16.7)
Big cities (≥100,000 inhabitants)	130,179 (14.3)	3923 (16.3)
Others	650,027 (71.2)	16,071 (66.9)
Smoking during pregnancy^a^		
No	481,730 (78.4)	18,637 (81.9)
Yes	111,475 (18.1)	3535 (15.5)
Missing	21,622 (3.5)	588 (2.6)
Charlson comorbidity index ≥1	14,483 (1.6)	1391 (5.8)
Gestational hypertensive disorders	57,192 (6.3)	1749 (7.3)
Gestational diabetes mellitus	9427 (1.0)	518 (2.2)
Children characteristics		
Male	469,166 (51.4)	12,222 (50.9)
Premature birth	50,858 (5.6)	1571 (6.5)
Low birth weight	44,442 (4.9)	1223 (5.1)
Five‐minute Apgar score <7	9252 (1.0)	227 (0.9)
Neonatal mortality	4375 (0.5)	64 (0.3)

^a^ Smoking during pregnancy was available from 1991 to 2016.

**FIGURE 1:**
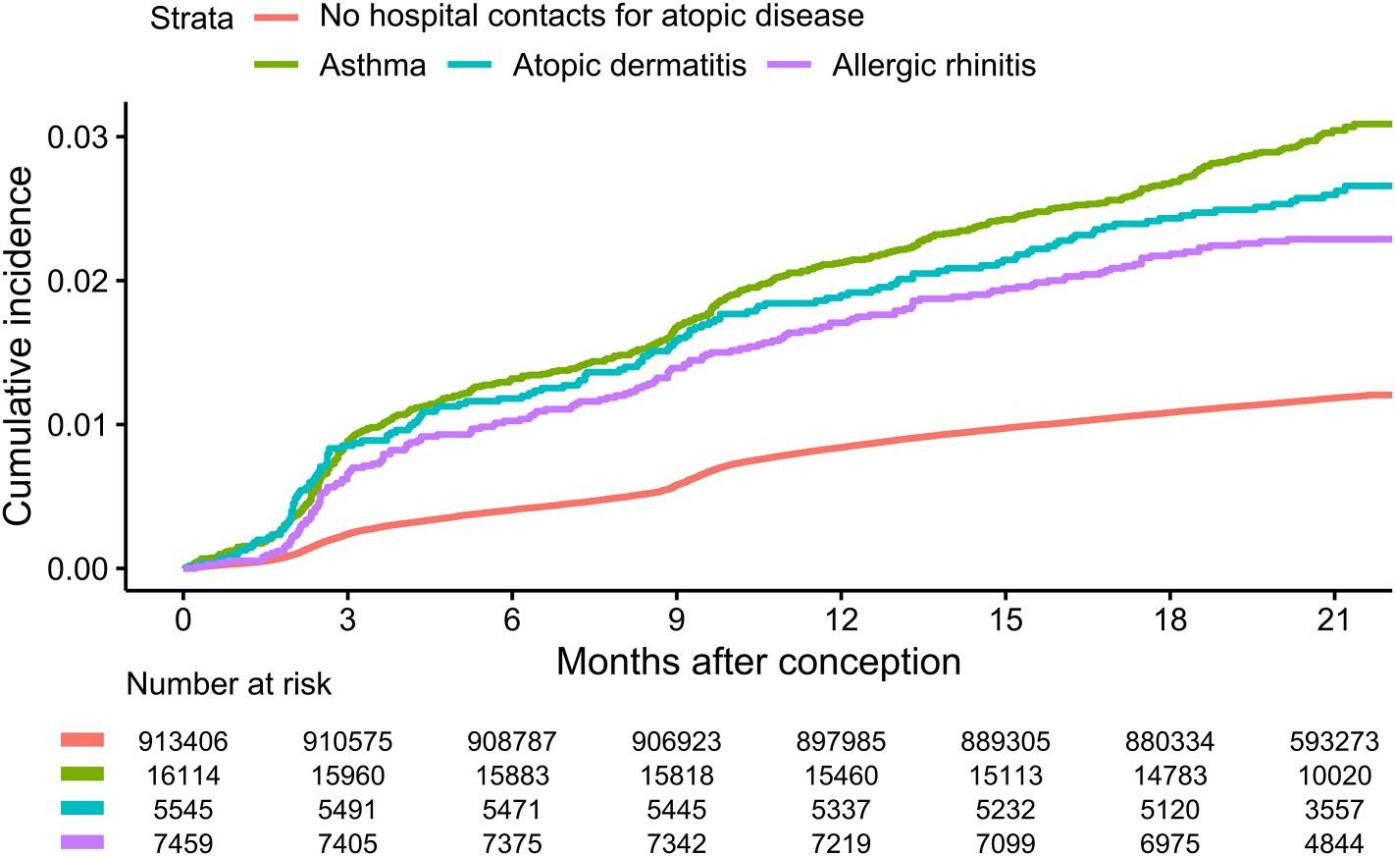
Cumulative incidence for peripartum mental disorders in women with hospital contacts for asthma, atopic dermatitis, allergic rhinitis, and without the 3 atopic diseases. Patients with multiple types of atopic diseases enters multiple corresponding categories

Peripartum mental disorders affected 2.7% of the women exposed to asthma, atopic dermatitis, or allergic rhinitis, whereas only 1.2% of the unexposed were affected. After adjustment for potential confounders, we observed a 37% increased risk in exposed women (HR, 1.37; 95% CI, 1.27–1.49; Figures 1 and S2). The same pattern was observed for mental disorders diagnosed either during pregnancy or postpartum (Table 2). Subgroup analyses revealed that the risk was more eminent during pregnancy than postpartum (Table 2). Sensitivity analyses, including both primiparous and multiparous women, and women with favorable pregnancy outcomes, yielded similar results as the main analysis (Table S2). In a subanalysis incorporating medication data to define atopic diseases after 1996, the results were similar with our main analysis, though the magnitude of association was less pronounced (HR, 1.22; 95% CI, 1.17–1.27; Table S3). The onset age didn't essentially alter the associations (Table S4). A more pronounced association was observed in women giving birth in spring (HR, 1.49; 95% CI, 1.27–1.75), while the association attenuated in women giving birth in summer (HR, 1.29; 95% CI, 1.10–1.51; Table S5).

**TABLE 2:** Association between asthma, atopic dermatitis, and allergic dermatitis before pregnancy and peripartum mental disorders (*n* = 937,422)

Exposure and outcome	No. of newly onset mental disorders (%)	Incidence per 1000 person‐years	Crude HR (95% CI)^a^	Adjusted HR (95% CI)^b^
Peripartum mental disorders				
No hospital contacts for atopic disease	10,709 (1.2)	6.8	1.0 (ref)	1.0 (ref)
Atopic disease	647 (2.7)	15.9	1.44 (1.33–1.56)	1.37 (1.27–1.49)
Asthma	476 (3.0)	17.5	1.55 (1.41–1.70)	1.44 (1.31–1.58)
Atopic dermatitis	142 (2.6)	15.1	1.39 (1.18–1.64)	1.36 (1.15–1.61)
Allergic rhinitis	167 (2.2)	13.1	1.24 (1.06–1.44)	1.30 (1.11–1.51)
Mental disorders during pregnancy				
No hospital contacts for atopic disease	5644 (0.6)	8.1	1.0 (ref)	1.0 (ref)
Atopic disease	377 (1.6)	20.8	1.45 (1.31–1.61)	1.37 (1.23–1.52)
Asthma	271 (1.7)	22.3	1.51 (1.34–1.71)	1.38 (1.22–1.56)
Atopic dermatitis	93 (1.7)	22.2	1.57 (1.28–1.93)	1.54 (1.25–1.89)
Allergic rhinitis	103 (1.4)	18.3	1.34 (1.10–1.63)	1.42 (1.16–1.72)
Postpartum mental disorders				
No hospital contacts for atopic disease	5065 (0.6)	5.7	1.0 (ref)	1.0 (ref)
Atopic disease	270 (1.1)	11.9	1.42 (1.26–1.61)	1.37 (1.21–1.55)
Asthma	205 (1.3)	13.6	1.60 (1.39–1.84)	1.50 (1.30–1.72)
Atopic dermatitis	49 (0.9)	9.4	1.13 (0.85–1.50)	1.11 (0.84–1.47)
Allergic rhinitis	64 (0.9)	9.0	1.10 (0.86‐1.40)	1.14 (0.89–1.46)

*Note*: Patients with multiple types of atopic diseases enters multiple corresponding subgroups, thus the numbers of each atopic disease did not sum to the total “atopic diseases”.

Abbreviations: CI, confidence interval; HR, hazard ratio; ref, reference.

^a^ Adjusted for calendar year.

^b^ Adjusted for calendar year, age at childbirth, education level, residence, and Charlson comorbidity index.

The risk of peripartum mental disorders was slightly higher in the women with atopic multimorbidity (HR, 1.48; 95% CI, 1.23–1.77; Table 3). More frequent hospital contacts of asthma, atopic dermatitis, or allergic rhinitis were associated with a higher risk (HR for five or more times of contact, 1.80; 95% CI, 1.37–2.35; *p* for trend <0.0001; Tables 3,  S6, and S7). A recent hospital contact of an atopic disease before conception was associated with a higher risk (HR for diagnosis within 2 years, 1.74; 95% CI, 1.48–2.06; *p* for trend <0.0001; Tables 3 and S7).

**TABLE 3:** Association between the severity of atopic diseases before pregnancy and peripartum mental disorders (*n* = 937,422)

Exposure	No. of newly onset mental disorders (%)	Incidence per 1000 person‐years	Crude HR (95% CI)^a^	Adjusted HR (95% CI)^b^	*p* for trend
Atopic multimorbidity					–
No	527 (2.7)	15.9	1.44 (1.31–1.57)	1.35 (1.24–1.47)	
Yes	120 (2.7)	15.8	1.45 (1.21–1.74)	1.48 (1.23–1.77)	
Number of hospital contacts for atopic disease					<0.0001
1	355 (2.6)	15.0	1.38 (1.24–1.53)	1.32 (1.19–1.47)	
2–4	241 (2.8)	16.3	1.44 (1.27–1.64)	1.38 (1.22–1.57)	
5 or more	51 (3.6)	21.5	2.02 (1.54–2.65)	1.80 (1.37–2.35)	
Period since last hospital contact for atopic diseases to conception					<0.0001
<2 years	140 (3.1)	18.4	1.91 (1.62–2.26)	1.74 (1.48–2.06)	
2–10 years	270 (2.6)	15.0	1.44 (1.27–1.62)	1.38 (1.22–1.56)	
>10 years	237 (2.6)	15.6	1.26 (1.10–1.43)	1.21 (1.07–1.38)	

Abbreviations: CI, confidence interval; HR, hazard ratio.

^a^ Adjusted for calendar year.

^b^ Adjusted for calendar year, age at childbirth, education level, residence, and Charlson comorbidity index.

**FIGURE 2:**
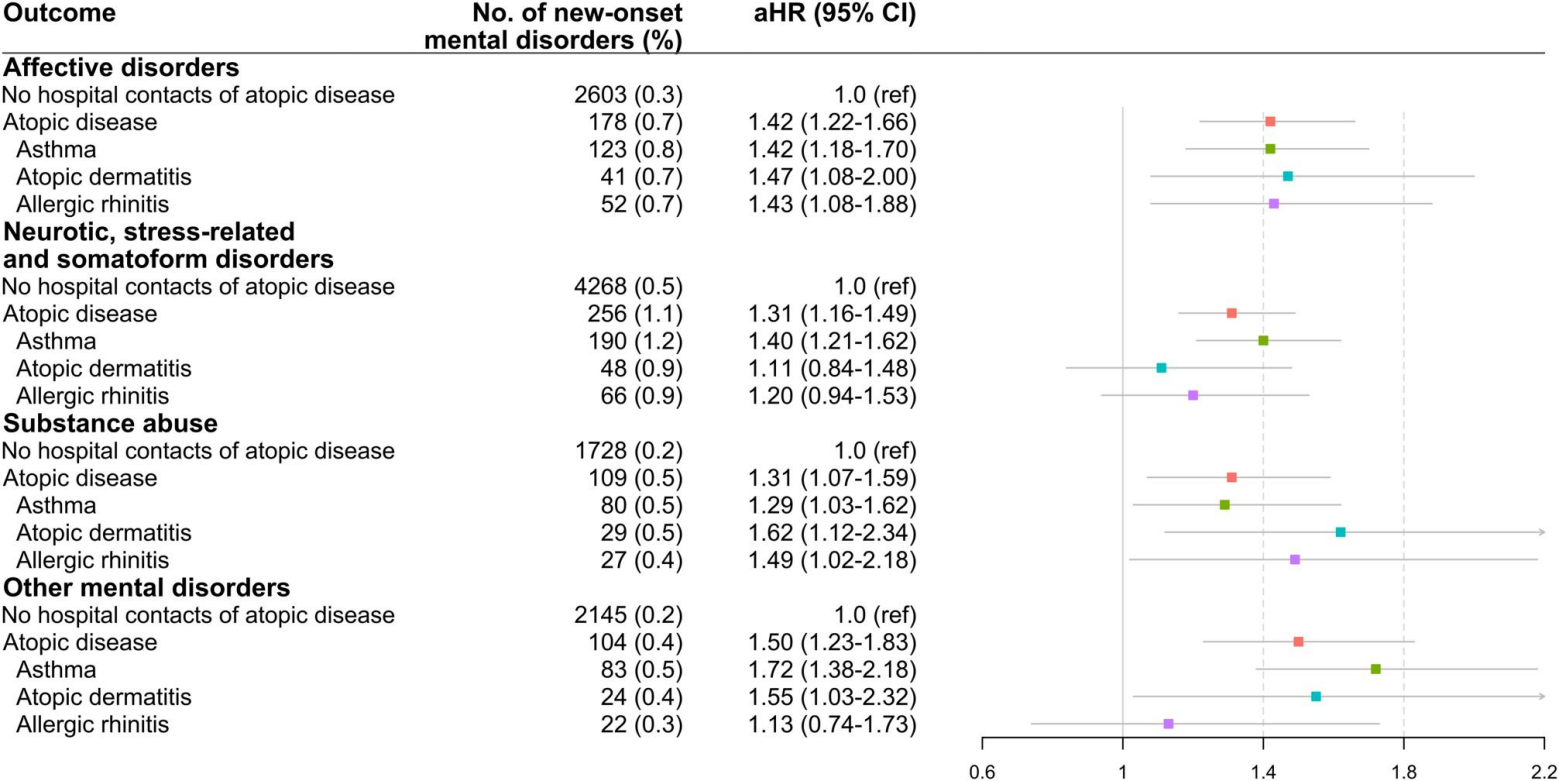
Association between hospital contacts for asthma, atopic dermatitis, and allergic dermatitis before pregnancy and different types of mental disorders during peripartum period (*n* = 937,422). Patients with multiple types of atopic diseases enters multiple corresponding subgroups, thus the numbers of each atopic disease did not sum to the total “atopic diseases”. aHRs were adjusted for calendar year, age at childbirth, education level, residence, and Charlson comorbidity index. The most important diagnostic entities contributing to “other mental disorders”: unspecified type of mental disorder (ICD‐10, F99), personality disorder (ICD‐10, F60), and mental and behavioral disorders associated with the puerperium (ICD‐10, F53). aHR, adjusted hazard ratio; CI, confidence interval; ref, reference

Overall, atopic disease (asthma, atopic dermatitis, and allergic rhinitis) was associated with increased risks of the three psychiatric diagnostic groups, namely affective disorders (HR, 1.42%; 95%, CI 1.22–1.66), neurotic, stress‐related and somatoform disorders (HR, 1.31; 95% CI, 1.16–1.49), and substance abuse (HR, 1.31; 95% CI, 1.07–1.59), respectively (Figure 2). Asthma, atopic dermatitis, and allergic rhinitis were all associated with affective disorders at a similar level (Figure 2). In particular, asthma was associated with a higher risk of neurotic, stress‐related and somatoform disorders (HR 1.40; 95% CI, 1.21–1.62; Figure 2), atopic dermatitis with a higher risk of substance abuse (HR 1.62; 95% CI, 1.12–2.34; Figure 2).

## 4 DISCUSSION

In this population‐based cohort study, we observed an overall 37% increased risk of peripartum mental disorders associated with a before‐conception diagnosis of either of the three atopic diseases: asthma, atopic dermatitis, and allergic rhinitis. The risk was higher among women with atopic multimorbidity, recurrent atopic disease, and active atopic disease. All the three types of atopic diseases were associated with affective disorders, while strong association was found between asthma and neurotic, stress‐related and somatoform disorders, and between atopic dermatitis and substance abuse.

The association between pre‐pregnancy asthma and peripartum mental disorders was reported in two population‐based cohort studies, which found a 52% increased risk of depression during pregnancy and a 58% increased risk of depression postpartum, respectively.^16^, ^17^ Both studies only focused on pregnancy‐related depression. Using this large population‐based cohort, we observed that women with a diagnosis of asthma, atopic dermatitis, and allergic rhinitis before conception had a higher overall risk of newly onset peripartum mental disorders. Atopic diseases are often poorly controlled during peripartum period, due to the systematically altered immune status and the non‐adherence to pharmacological treatment in peripartum women.^34^, ^35^ As a result, aggravated symptoms and atopy may influence mental disorders on peripartum period though a number of causal pathways. Aggravated atopic symptoms may contribute to mental stress, through sleep disorder induced by severe pruritus or dyspnea.^34^, ^36^ Also, allergic reactions may dysregulate autoimmune system, which might trigger following mental disorders.^37^ The peripheral inflammation ignited from skin, nasal cavities, and respiratory tract may affect the brain, transmitted by dysregulated inflammatory cytokines, such as interleukin‐6, interleukin‐1β, and tumor necrosis factor.^38^, ^39^ These mechanisms may explain and support our findings of a higher risk of peripartum mental disorders in women with more frequent or recent hospital contacts for atopic diseases. In addition, shared genetic susceptibility may be part of the underlying causes given the genetic overlaps between asthma and attention deficit hyperactivity disorder, anxiety, and major depressive disorder.^40^

Currently, most studies on peripartum mental disorders have focused on depression, even though anxiety is more prevalent than depression during the peripartum period.^30^ Our analyses found that peripartum neurotic, stress‐related and somatoform disorders, mainly comprised of anxiety, were specifically associated with asthma. This pattern has been recognized in the general population, partially due to poor asthma control.^41^, ^42^ It also suggests a potentially different mechanism underlying the association between asthma and anxiety from that for the association between atopic disease and depression. This is possibly due to the significant heterogeneity in immunopathology in asthma, compared with the generally consistent characteristic in atopic dermatitis and allergic rhinitis (immune imbalance toward a T‐helper‐2 response).^43^, ^44^, ^45^ In addition, we observed a novel association between atopic dermatitis and peripartum substance abuse. Similar results were reported in the general Danish population, where the prevalence of smoking and alcohol abuse was higher among atopic dermatitis patients.^46^

Effective management of peripartum mental disorders requires early detection.^47^, ^48^ Despite regular medical contacts during pregnancy and postpartum period in many countries, less than 20% of depressed parturients reports their symptoms to medical professionals.^48^ Our results suggest that women who have poor control of atopic diseases during pregnancy may benefit from systematic mental health monitoring and consultation with a specialist. Our study had several important strengths. First, we use a full population‐based design with high quality of data that minimize selection and recall bias. Second, the large sample size enabled us to explore specific associations for each pair of exposure and outcome. In particular, this study focused on the risks of a spectrum of mental disorders during the whole peripartum period, expanding the scope of both exposures and outcomes on this topic.

Our findings should also be interpreted in the light of the following limitations. First, the incidence of 1.2% for peripartum mental disorders in this study was lower than the reported incidence of ∼10% worldwide.^49^ Also, the prevalence of the three atopic diseases of interest was lower than that was reported in many areas.^9^ However, similar low incidence and prevalence were reported in other Nordic registry‐based studies with a high validity of diagnosis from hospital discharges, given the fact that patients with mild symptoms might not always seek medical care.^13^, ^50^, ^51^ We performed an additional sub‐analysis utilizing medication data to define atopic diseases, which was validated to increase the diagnostic sensitivity.^52^ The association persisted, while the risk estimate was a little lower. We cannot rule out the possibility of misclassification in both exposure and outcomes, in which case the observed elevated risks would be overestimated and reflect the associations in relation to severe cases of atopic diseases and the outcome of interest. Second, the severity of atopic diseases was not able to be directly measured by symptoms or biological indicators because such data were not available in the registers. Also, disease severity may differ in patients taking medication or other treatment, which were not considered in our analysis. On the other hand, this study defined the severity of atopy with hospital‐based data with three approaches to measure, all showing similarly that severe atopy was associated with a higher risk of peripartum mental disorders. Third, patients with atopic diseases were more likely to seek medical advice, thus more likely to undergo psychotic screening. However, during the peripartum period, most pregnant women pay regular visits to midwives and doctors, thus selection bias might be less likely. Furthermore, we included comorbidity as a covariate to adjust for such confounding. Fourth, other atopic diseases, like food allergy, could not be characterized by the ICD‐code‐based diagnoses. This may cause misclassification bias and led to an underestimation of the association between atopic diseases and peripartum mental disorders. However, the studied three types represent the major atopic diseases.^9^ Fifth, there was a latency period between the date of diagnosis from hospital and actual disease onset, thus the timing of outcomes (during pregnancy and postpartum) should be interpreted with caution. Sixth, as we used hospital‐based data to identify and exclude women with a history of mental disorder, mild cases with no hospital contacts were still included in the study population. When diagnosed during peripartum period, these patients with mild mental disorders would be misclassified as peripartum mental disorders. This could result in an overestimation of the incidence of peripartum mental disorders.

## 5 CONCLUSION

History of asthma, atopic dermatitis, and allergic rhinitis were associated with increased risks of peripartum mental disorders. All the three types of atopic diseases were associated with affective disorders. Strong associations were observed between asthma and neurotic, stress‐related and somatoform disorders, and between atopic dermatitis and substance abuse. Women who have atopic diseases before pregnancy may benefit from future systematic mental health monitoring.

## CONFLICT OF INTEREST

The authors have no conflict of interests.

## AUTHOR CONTRIBUTIONS

Tai Ren and Jiawen Chen contributed equally to this work as co‐first authors. Tai Ren, Jiawen Chen, and Jiong Li conceived the study. Fei Li, Hua He, Carsten Obel, Katrine Svendsen, Jun Zhang, and Jiong Li contributed to the design. Katrine Svendsen, Carsten Obel, and Jiong Li recruited the data. Tai Ren, Yongfu Yu, and Hui Wang undertook the statistical analysis. Tai Ren and Jiawen Chen drafted the first draft of the manuscript. All authors provided critical input to the analyses, interpreted the data, and revised the manuscript. The corresponding author confirms that all listed authors meet authorship criteria and that no others meeting the criteria have been omitted.

## Supporting information

Supporting Information S1
