# Supplementary material for: The association of asthma, atopic dermatitis, and allergic rhinitis with peripartum mental disorders
Source: Clin Transl Allergy. 2021 Dec 3;11(10):e12082. doi: 10.1002/clt2.12082 (PMC8805685; doi:10.1002/clt2.12082)
Supplement: Supplementary file 1 — Supporting Information S1 [file CLT2-11-e12082-s001.docx]

**The association of asthma, atopic dermatitis, and allergic rhinitis with** **peripartum mental disorders**

**Supplementary Data**

Tai Ren, Jiawen Chen, Yongfu Yu, Hua He, Jun Zhang, Fei Li, Katrine Svendsen, Carsten Obel, Hui Wang, Jiong Li

**Table of Contents**

Supplementary

[Supplementary Table S1 Diagnosis codes for Charlson comorbidity index and its score 3](#_Toc86053351)

[Supplementary Table S2 Sensitivity analyses of the association between atopic diseases before pregnancy and new-onset peripartum mental disorders. 4](#_Toc86053352)

[Supplementary Table S3 Association between asthma, atopic dermatitis, and allergic rhinoconjunctivitis before pregnancy and peripartum mental disorders, with atopic diseases defined by both hospital contacts and disease-specific medications, 1996-2016 (n=496 224). 6](#_Toc86053353)

[Supplementary Table S4 Association between onset age of atopic diseases and peripartum mental disorders (n=937 422). 7](#_Toc86053354)

[Supplementary Table S5 Association between asthma, atopic dermatitis, and allergic rhinitis before pregnancy and peripartum mental disorders, according to season of the childbirth (n=937 422). 8](#_Toc86053355)

[Supplementary Table S6 Association between asthma, atopic dermatitis, and allergic rhinitis before pregnancy and peripartum mental disorders, according to the number of hospital contacts for the three different atopic diseases (n=937 422). 9](#_Toc86053356)

[Supplementary Table S7 Association between the severity of atopic diseases before pregnancy and peripartum mental disorders, with different weights assigned to each category (n=937 422). 10](#_Toc86053357)

[Supplementary Figure S1 Directed acyclic graph showing selection of covariates for confounding control. 11](#_Toc86053358)

[Supplementary Figure S2 Log[− log S(t)] plot for women with and without atopic diseases before conception. 12](#_Toc86053359)

[Supplementary Figure S3 Cumulative incidence for peripartum mental disorders in women with and without atopic diseases before conception. 13](#_Toc86053360)

[Supplementary Appendix S1 Methods used to define atopic diseases in a subanalysis, in order to increase the diagnostic sensitivity. 14](#_Toc86053361)

# Supplementary Table S1 Diagnosis codes for Charlson comorbidity index and its score

| **Charlson comorbidity category** | **ICD–8**  **(1978–1993)** | **ICD–10**  **(since 1994)** | **Charlson comorbidity index score** |
| --- | --- | --- | --- |
| Myocardial infarction | 410 | I21; I22; I23 | 1 |
| Congestive heart failure | 427.09; 427.10; 427.11; 427.19; 428.99; 782.49 | I50; I11.0; I13.0; I13.2 | 1 |
| Peripheral vascular disease | 440; 441; 442; 443; 444; 445 | I70; I71; I72; I73; I74; I77 | 1 |
| Cerebrovascular disease | 430–438 | I60–I69; G45; G46 | 1 |
| Dementia | 290.09–290.19; 293.09 | F00–F03; F05.1; G30 | 1 |
| Chronic pulmonary disease | 490–493; 515–518;  excluding 493 | J40–J47; J60–J67; J68.4; J70.1; J70.3; J84.1; J92.0; J96.1; J98.2; J98.3;  excluding J45-J46 | 1 |
| Connective tissue disease | 712; 716; 734; 446; 135.99 | M05; M06; M08; M09; M30; M31; M32; M33; M34; M35; M36; D86 | 1 |
| Ulcer disease | 530.91; 530.98; 531–534 | K22.1; K25–K28 | 1 |
| Mild liver disease | 571; 573.01; 573.04 | B18; K70.0–K70.3; K70.9; K71; K73; K74; K76.0 | 1 |
| Diabetes | 249.00; 249.06; 249.07; 249.09; 250.00; 250.06; 250.07; 250.09 | E10.0, E10.1; E10.9; E11.0; E11.1; E11.9 | 1 |
| Hemiplegia | 344 | G81; G82 | 2 |
| Moderate to severe renal disease | 403; 404; 580–583; 584; 590.09; 593.19; 753.10–753.19; 792 | I12; I13; N00–N05; N07; N11; N14; N17–N19; Q61 | 2 |
| Diabetes with end organ damage | 249.01–249.05; 249.08; 250.01–250.05; 250.08 | E10.2–E10.8; E11.2–E11.8 | 2 |
| Any tumor | 140–194; 204–207 | C00–C75; C91–C95 | 2 |
| Lymphoma | 200–203; 275.59 | C81–C85; C88; C90; C96 | 2 |
| Moderate to severe liver disease | 070.00; 070.02; 070.04; 070.06; 070.08; 573.00; 456.00–456.09 | B15.0; B16.0; B16.2; B19.0; K70.4; K72; K76.6; I85 | 3 |
| Metastatic solid tumor | 195–198; 199 | C76–C80 | 6 |
| AIDS | 079.83 | B21–B24 | 6 |

# Supplementary Table S2 Sensitivity analyses of the association between atopic diseases before pregnancy and new-onset peripartum mental disorders.

| **Analysis** | **Exposure** | **No. of new-onset mental disorders (%)** | **Incidence per 1000 person-years** | **Adjusted HR (95% CI)^a^** |
| --- | --- | --- | --- | --- |
| **Additional adjustment of maternal smoking (n=637 587)** | No hospital contacts for atopic disease | 9585 (1.6) | 9.1 | 1.0 (ref) |
|  | Atopic disease | 641 (2.8) | 16.6 | 1.37 (1.26-1.48) |
|  | Asthma | 474 (3.1) | 18.3 | 1.42 (1.29-1.56) |
|  | Atopic Dermatitis | 139 (2.6) | 15.6 | 1.32 (1.12-1.56) |
|  | Allergic Rhinitis | 164 (2.3) | 13.7 | 1.30 (1.12-1.52) |
| **Excluding women diagnosed as atopic disease from conception to 2-year postpartum (n=888 044)** | No hospital contacts for atopic disease | 9680 (1.1) | 6.4 | 1.0 (ref) |
|  | Atopic disease | 556 (2.6) | 15.3 | 1.38 (1.27-1.51) |
|  | Asthma | 406 (2.9) | 16.8 | 1.44 (1.30-1.59) |
|  | Atopic Dermatitis | 124 (2.5) | 14.6 | 1.35 (1.13-1.62) |
|  | Allergic Rhinitis | 148 (2.2) | 12.7 | 1.30 (1.10-1.53) |
| **Pregnancies after 1994^b^ (n=552 953)** | No hospital contacts for atopic disease | 9314 (1.8) | 10.2 | 1.0 (ref) |
|  | Atopic disease | 635 (2.9) | 17.1 | 1.36 (1.25-1.47) |
|  | Asthma | 468 (3.1) | 18.6 | 1.40 (1.28-1.54) |
|  | Atopic Dermatitis | 139 (2.7) | 16.2 | 1.33 (1.12-1.57) |
|  | Allergic Rhinitis | 163 (2.4) | 14.2 | 1.31 (1.12-1.52) |
| **Additional inclusion of multiparous women^c^ (n=1 112 787)** | No hospital contacts for atopic disease | 16472 (1.5) | 8.8 | 1.0 (ref) |
|  | Atopic disease | 938 (3.4) | 20.1 | 1.37 (1.28-1.47) |
|  | Asthma | 687 (3.6) | 21.7 | 1.42 (1.32-1.54) |
|  | Atopic Dermatitis | 208 (3.4) | 20.5 | 1.42 (1.24-1.63) |
|  | Allergic Rhinitis | 241 (2.8) | 16.7 | 1.28 (1.12-1.45) |
| **5-min Apgar ≥7, excluding pre-term, low birth weight, neonatal mortality (n=851 695)** | No hospital contacts for atopic disease | 9266 (1.1) | 6.4 | 1.0 (ref) |
|  | Atopic disease | 556 (2.6) | 15.1 | 1.36 (1.25-1.49) |
|  | Asthma | 408 (2.8) | 16.6 | 1.42 (1.28-1.57) |
|  | Atopic Dermatitis | 123 (2.4) | 14.4 | 1.34 (1.12-1.60) |
|  | Allergic Rhinitis | 142 (2.1) | 12.3 | 1.25 (1.06-1.48) |
| **Exposure as a time-varying variable (n=937 422)** | No hospital contacts for atopic disease | — | 6.8 | 1.0 (ref) |
|  | Atopic disease | — | 16.6 | 1.37 (1.27-1.48) |
|  | Asthma | — | 18.3 | 1.43 (1.31-1.56) |
|  | Atopic Dermatitis | — | 15.6 | 1.37 (1.16-1.61) |
|  | Allergic Rhinitis | — | 13.1 | 1.28 (1.10-1.50) |

HR, hazard ratio; CI, confidence interval; ref, reference; patients with multiple types of atopic diseases enters multiple corresponding subgroups, thus the numbers of each atopic disease did not sum to the total “atopic diseases”.

^a^ Adjusted for calendar year, age at childbirth, education level, residence, and Charlson comorbidity index.

^b^ The HR was estimated additionally adjusted for maternal smoking.

^c^ The HR was estimated additionally adjusted for parity.

# Supplementary Table S3 Association between asthma, atopic dermatitis, and allergic rhinoconjunctivitis before pregnancy and peripartum mental disorders, with atopic diseases defined by both hospital contacts and disease-specific medications, 1996-2016 (n=496 224).

| **Exposure** | **No. of newly-onset mental disorders (%)** | **Incidence per 1000 person-years** | **Crude HR  (95% CI)^a^** | **Adjusted HR (95% CI)^b^** |
| --- | --- | --- | --- | --- |
| No hospital contacts for atopic disease | 6132 (1.7) | 10.1 | 1.0 (ref) | 1.0 (ref) |
| Atopic disease | 3364 (2.3) | 13.8 | 1.20 (1.15-1.25) | 1.22 (1.17-1.27) |
| Asthma | 717 (2.8) | 16.7 | 1.41 (1.31-1.53) | 1.34 (1.24-1.45) |
| Atopic dermatitis | 1702 (2.4) | 14.3 | 1.23 (1.16-1.30) | 1.23 (1.17-1.30) |
| Allergic rhinoconjunctivitis | 2020 (2.3) | 13.6 | 1.18 (1.13-1.25) | 1.22 (1.16-1.28) |

^a^ Adjusted for calendar year.

^b^ Adjusted for calendar year, age at childbirth, education level, residence, and Charlson comorbidity index.

# Supplementary Table S4 Association between onset age of atopic diseases and peripartum mental disorders (n=937 422).

| **Exposure** | **No. of newly -onset mental disorders (%)** | **Incidence per 1000 person-years** | **Adjusted HR (95% CI)^a^** |
| --- | --- | --- | --- |
| **Age at first diagnosis of atopic diseases** |  |  |  |
| <10 yo | 202 (3.3) | 19.5 | 1.37 (1.19-1.58) |
| 10-20 yo | 215 (2.5) | 14.4 | 1.28 (1.11-1.46) |
| >20 yo | 230 (2.5) | 14.9 | 1.48 (1.30-1.69) |

HR, hazard ratio; CI, confidence interval; ref, reference;

^a^ Adjusted for calendar year, age at childbirth, education level, residence, and Charlson comorbidity index.

# Supplementary Table S5 Association between asthma, atopic dermatitis, and allergic rhinitis before pregnancy and peripartum mental disorders, according to season of the childbirth (n=937 422).

| **Season of childbirth** | **Exposure** | **No. of new-onset mental disorders (%)** | **Incidence per 1000 person-years** | **Adjusted HR (95% CI)^a^** |
| --- | --- | --- | --- | --- |
| **Spring** | No hospital contacts for atopic disease | 2588 (1.1) | 6.6 | 1.0 (ref) |
|  | Atopic disease | 163 (2.9) | 17.0 | 1.49 (1.27-1.75) |
|  | Asthma | 126 (3.3) | 19.6 | 1.63 (1.36-1.95) |
|  | Atopic Dermatitis | 25 (2.0) | 11.5 | 1.05 (0.71-1.56) |
|  | Allergic Rhinitis | 40 (2.3) | 13.1 | 1.32 (0.97-1.81) |
| **Summer** | No hospital contacts for atopic disease | 2821 (1.2) | 6.7 | 1.0 (ref) |
|  | Atopic disease | 163 (2.5) | 14.7 | 1.29 (1.10-1.51) |
|  | Asthma | 113 (2.6) | 15.3 | 1.27 (1.05-1.54) |
|  | Atopic Dermatitis | 42 (2.7) | 16.2 | 1.16 (0.85-1.58) |
|  | Allergic Rhinitis | 40 (2.0) | 11.6 | 1.38 (1.17-1.62) |
| **Autumn** | No hospital contacts for atopic disease | 2708 (1.2) | 6.9 | 1.0 (ref) |
|  | Atopic disease | 163 (2.7) | 15.9 | 1.34 (1.15-1.58) |
|  | Asthma | 122 (3.0) | 17.7 | 1.43 (1.19-1.71) |
|  | Atopic Dermatitis | 36 (2.6) | 15.4 | 1.36 (0.98-1.89) |
|  | Allergic Rhinitis | 39 (2.1) | 12.3 | 1.21 (0.88-1.66) |
| **Winter** | No hospital contacts for atopic disease | 2592 (1.2) | 6.9 | 1.0 (ref) |
|  | Atopic disease | 158 (2.8) | 16.2 | 1.38 (1.17-1.62) |
|  | Asthma | 115 (3.0) | 17.5 | 1.42 (1.18-1.75) |
|  | Atopic Dermatitis | 39 (2.9) | 16.9 | 1.50 (1.09-2.06) |
|  | Allergic Rhinitis | 48 (2.7) | 15.7 | 1.51 (1.13-2.01) |

HR, hazard ratio; CI, confidence interval; ref, reference;

^a^ Adjusted for calendar year, age at childbirth, education level, residence, and Charlson comorbidity index.

# Supplementary Table S6 Association between asthma, atopic dermatitis, and allergic rhinitis before pregnancy and peripartum mental disorders, according to the number of hospital contacts for the three different atopic diseases (n=937 422).

| **Exposure** | **No. of newly -onset mental disorders (%)** | **Incidence per 1000 person-years** | **Crude HR (95% CI)^a^** | **Adjusted HR (95% CI)^b^** | **P for trend^c^** |
| --- | --- | --- | --- | --- | --- |
| **No. hospital contacts for asthma** |  |  |  |  | <0.0001 |
| 1 | 225 (2.9) | 17.4 | 1.54 (1.35-1.76) | 1.35 (1.19-1.47) |  |
| 2~4 | 403 (5.7) | 33.4 | 1.48 (1.28-1.70) | 1.38 (1.22-1.57) |  |
| 5 or more | 48 (3.6) | 21.6 | 2.04 (1.54-2.69) | 1.80 (1.37-2.35) |  |
| **No. hospital contacts for atopic dermatitis** |  |  |  |  | <0.0001 |
| 1 | 64 (2.4) | 13.8 | 1.28 (1.00-1.63) | 1.25 (0.98-1.60) |  |
| 2~4 | 51 (2.4) | 13.9 | 1.28 (0.97-1.68) | 1.27 (0.96-1.67) |  |
| 5 or more | 27 (4.1) | 24.6 | 2.45 (1.71-3.51) | 2.30 (1.60-3.29) |  |
| **No. hospital contacts for allergic rhinitis** |  |  |  |  | <0.0001 |
| 1 | 66 (1.9) | 11.0 | 1.08 (0.85-1.38) | 1.14 (0.90-1.45) |  |
| 2~4 | 80 (2.4) | 14.0 | 1.29 (1.04-1.61) | 1.37 (1.10-1.70) |  |
| 5 or more | 21 (3.4) | 20.0 | 1.77 (1.16-2.71) | 1.72 (1.12-2.62) |  |

HR, hazard ratio; CI, confidence interval; ref, reference;

^a^ Adjusted for calendar year.

^b^ Adjusted for calendar year, age at childbirth, education level, residence, and Charlson comorbidity index.

^c^ Weights assigned according to the median of each category.

# Supplementary Table S7 Association between the severity of atopic diseases before pregnancy and peripartum mental disorders, with different weights assigned to each category (n=937 422).

| **Exposure** | **Adjusted HR (95% CI)^a^** | **Model 1** | | **Model 2** | | **Model 3** | |
| --- | --- | --- | --- | --- | --- | --- | --- |
|  |  | **Weight assigned** | **P for trend** | **Weight assigned** | **P for trend** | **Weight assigned** | **P for trend** |
| **Numer of hospital contacts for atopic disease** |  |  | <0.0001 |  | <0.0001 |  | <0.0001 |
| 1 | 1.32 (1.19-1.47) | 1 |  | 1 |  | 1 |  |
| 2~4 | 1.38 (1.22-1.57) | 2 |  | 4 |  | 2 |  |
| 5 or more | 1.80 (1.37-2.35) | 8 |  | 8 |  | 5 |  |
| **Period since last hospital contact for atopic diseases to conception** |  |  | <0.0001 |  | <0.0001 |  | <0.0001 |
| <2 years | 1.74 (1.48-2.06) | 1 |  | 1 |  | 1 |  |
| 2~10 years | 1.38 (1.22-1.56) | 5 |  | 8 |  | 5 |  |
| >10 years | 1.21 (1.07-1.38) | 16 |  | 16 |  | 10 |  |

^a^ Adjusted for calendar year, age at childbirth, education level, residence, and Charlson comorbidity index.


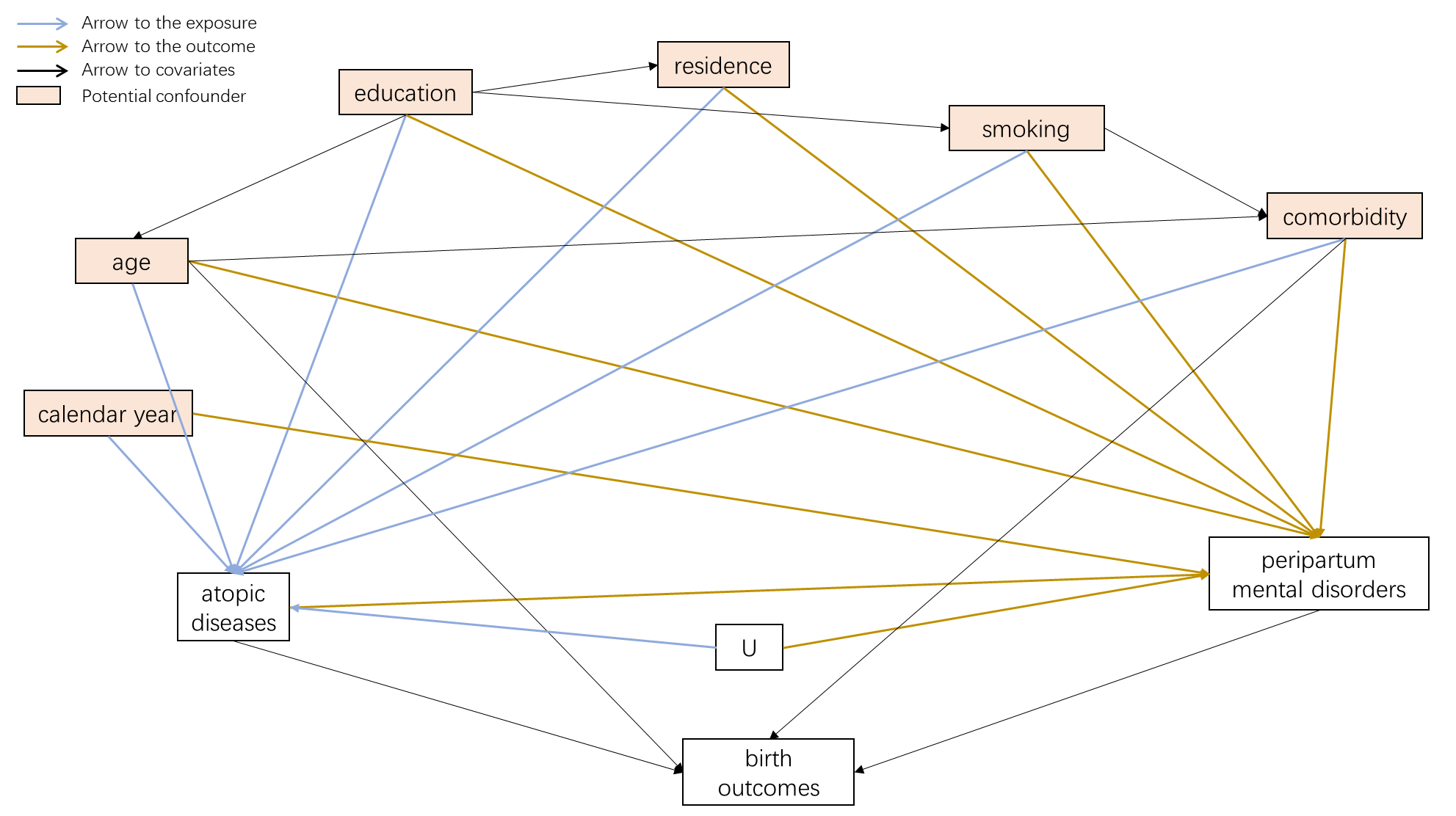


# Supplementary Figure S1 Directed acyclic graph showing selection of covariates for confounding control.

Note: the arrow from peripartum mental disorder to birth outcomes should be reversed for postpartum mental disorders, in which case birth outcomes could be mediators from atopic diseases to postpartum mental disorders.


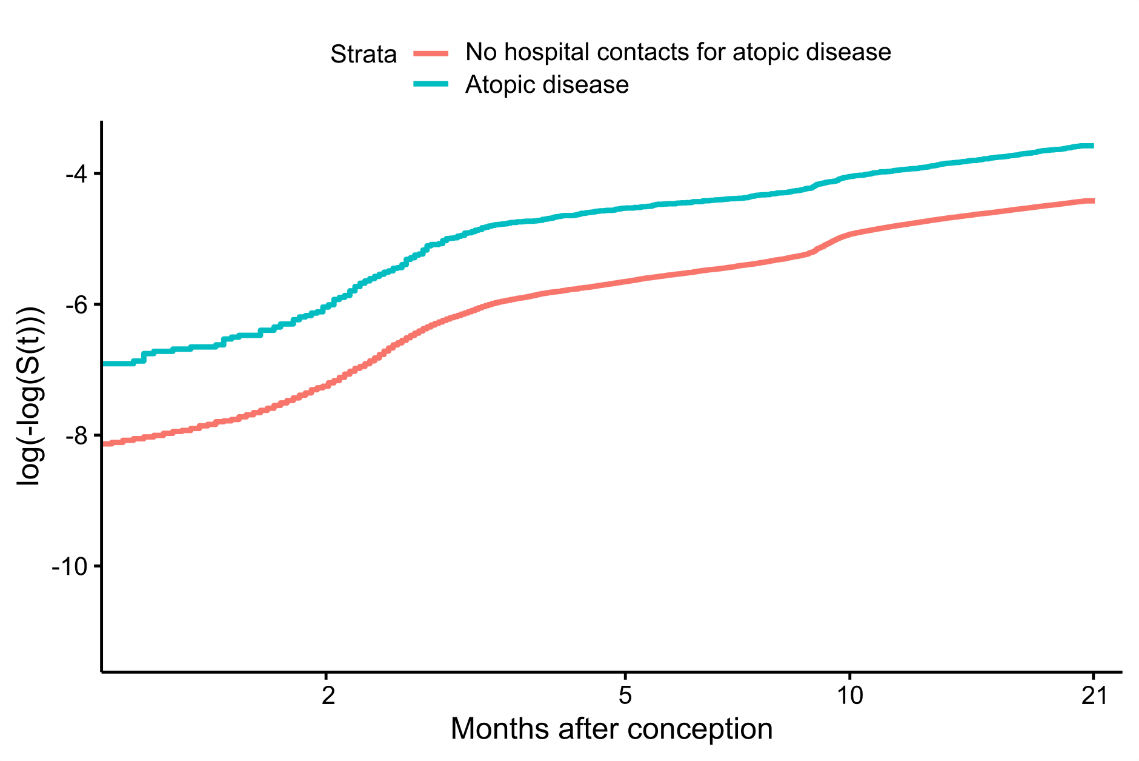


# Supplementary Figure S2 Log[− log S(t)] plot for women with and without atopic diseases before conception.


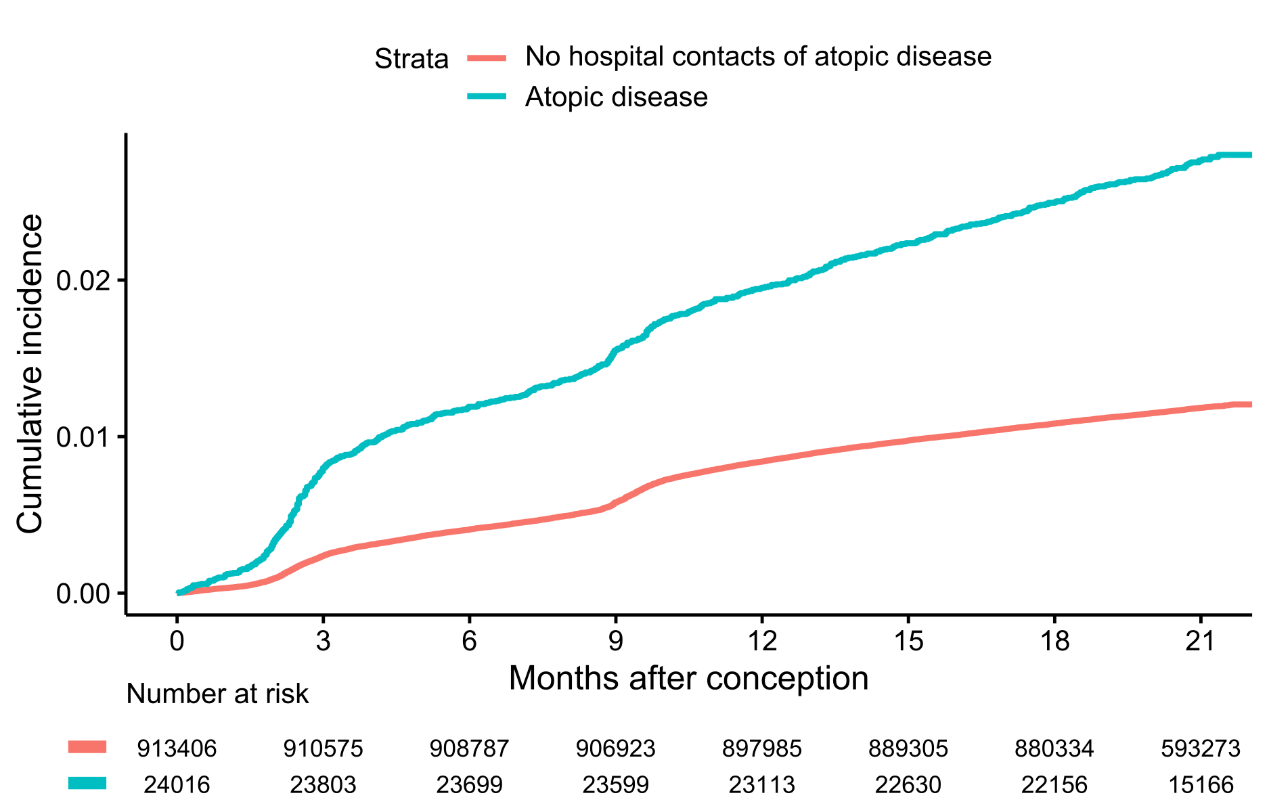


# Supplementary Figure S3 Cumulative incidence for peripartum mental disorders in women with and without atopic diseases before conception.

# Supplementary Appendix S1 Methods used to define atopic diseases in a subanalysis, in order to increase the diagnostic sensitivity.

Henriksen et al.^1^ proposed an algorithm to identify atopic dermatitis, asthma, and allergic rhinoconjunctivitis that utilized data from the Danish National Patient Register and the Danish National Prescription Registry. The algorithm was further validated in paediatric patients with sensitivities between 74% and 84% and specificities between 73% to 82%.^2^ The Danish National Prescription Registry contains information on all redeemed prescriptions dispensed in pharmacy in Denmark since 1995.^3^ In brief, the algorithms defined each of the three atopic diseases by three steps: 1) identify disease- specific hospital diagnoses and disease- specific medications; 2) given that atopic diseases are characterized by chronicity and recurrence, the disease-specific medications were required to be dispensed at least twice within a year; 3) exclude prescriptions that might be relevant to other medical conditions. To be noted, the algorithm defined allergic rhinoconjunctivitis rather than allergic rhinitis as in this study. Using the same algorithm, we investigated the association between asthma, atopic dermatitis, allergic rhinoconjunctivitis, defined with the new algorithm, and peripartum mental disorders in primiparous women who gave birth to a live-born between 1996 and 2016.

The detailed algorithm to define the three atopic diseases were as following^1^:

”**1. Algorithm to define patients with atopic dermatitis**

Patients with atopic dermatitis fulfilled either criterion 1 – 3.

**CRITERIA 1 (ICD-10):**

≥1 hospital contact for:

L20 “atopic dermatitis”

L308C “winter feet”

**CRITERIA 2 (based on ATC):**

≥1 filled prescription of: D11AH “agents for dermatitis: tacrolimus, pimecrolimus” **without any of the exclusion criteria specified below**

**CRITERIA 3 (based on ATC):**

≥ 2 filled prescriptions of: D07 “corticosteroids for topical use” within 12 months **without any of the exclusion criteria specified below**

Without co-occurring hospital contacts and/ or combination of filled prescriptions below (exclusions criteria): Children WITHOUT L20 “atopic dermatitis” with a diagnosis of: L21 “seborrhoeic dermatitis”, L22 “diaper dermatitis” L23 “allergic contact dermatitis”, L24 “irritant contact dermatitis”, L25 “unspecified contact dermatitis”, L26 “exfoliative dermatitis”, L27 “dermatitis due to substances taken internally”, L28 “lichen simplex chronicus and prurigo”, L29 “pruritus”, L30 “other dermatitis” (except L308C), L40 – L45 “papulosquamous disorders”, L53 “other erythematosus conditions”, L55 “sunburn”, L56 “other acute skin changes due to ultraviolet radiation”, L80 “vitiligo”, L90 “atrophic disorders of the skin”, L93 “lupus erythematosus”

OR/AND

**Exclusion medication criteria:**

≥1 filled prescription of either:

D05 “antipsoriasics” or D02AF “salicylates for dermatological use” or D07XB “corticosteroids moderate or potent other combinations“ or D07XC “corticosteroids moderate or potent other combinations“ or D07AD01 “corticosteroids (group IV) clobetasol” or D07CD01 “clobetasol and antibiotics”) AND D01 “antifungals” (implies corticosteroid (group IV) use for vaginal fungal infection)

*For prescription of corticosteroid group IV, a filled prescription of group I-III should also be used (as atopic dermatitis is never treated alone with group IV)

**2. Algorithm to define patients with asthma**

Patients with asthma fulfilled either criterion 1 and/or 2 below:

**CRITERIA 1 (based on ICD-10):**

≥1 hospital contact for:

J45.0”allergic asthma”

J45.1”non-allergic asthma”

J45.8 “asthma, different types”

J45.9 “asthma, unspecified”

J46.0 “status asthmaticus”

J46.9 “status asthmaticus, unspecified”

**CRITERIA 2 (based on ATC):**

> 1 filled prescription within 12 months of:

R03BA01 – R03BA08 “inhaled glucocorticoids”

R03DC01 – R03DC04 “leukotriene-receptor antagonists”

R03DC03 “montelukast (if no diagnosis of J30 allergic rhinitis)”

R03DB04 ”theophylline og adrenergics”

R03DA54 ”theophylline, combinations excl. psycholeptics”

R03BB01 ”anticholinergica, Ipratropium bromide”

R03DX05 ”omalizumab”

**3. Algorithm to define patients with allergic rhinoconjunctivitis**

Patients with allergic rhinoconjunctivitis fulfilled either criterion 1 – 4 below

**CRITERIA 1 (based on ICD-10):**

≥1 hospital contact for:

J30 “hay fever and allergic rhinitis”

J30.0 ”vasomotor rhinitis”

J30.1 “allergic rhinitis due to pollen”

J30.2 “other seasonal allergic rhinitis”

J30.3 “other allergic rhinitis”

J30.4 “allergic rhinitis, unspecified”

J31.0 “chronic rhinitis”

**CRITERIA 2 (based on ATC and ICD-10):**

≥ 2 filled prescriptions of:

R01AD01 – R01AD60 “inhaled corticosteroids for rhinitis”

And no hospital contact for (exclusions criteria):

J33 “nasal polyps”

J330 ”polyps in nasal cavity”

J331 “polyp related sinus degeneration”

J331A “woakes' ethmoiditis”

J338” nasal polyps, other”

J338A “polypus sinus sphenoidalis”

J339 “nasal polyps, unspecified”

J010- J019 ”acute sinusitis”

J320 –J329 ”chronic sinusitis”

**CRITERIA 3 (based on ICD-10 and ATC)**

≥2 filled prescriptions of:

R06A “antihistamines for systemic use”

And no hospital contact for:

L29 “pruritus” or

L50 “allergic urticaria”

**CRITERIA 4 (based on ATC)**

≥ 1 filled prescriptions of:

V01A “specific immune therapy, allergen substract therapy” or/and

S01GX “medication for allergic conjunctivitis” ”

**References:**

1. Henriksen L, Simonsen J, Haerskjold A, et al. Incidence rates of atopic dermatitis, asthma, and allergic rhinoconjunctivitis in Danish and Swedish children. *J Allergy Clin Immunol* 2015;136(2):360-6 e2.

2. Stensballe LG, Klanso L, Jensen A, et al. The validity of register data to identify children with atopic dermatitis, asthma or allergic rhinoconjunctivitis. *Pediatr Allergy Immunol* 2017;28(6):535-42.

3. Kildemoes HW, Sorensen HT, Hallas J. The Danish National Prescription Registry. *Scand J Public Health* 2011;39:38-41.
